# Effect of Displacement Degree of Distal Chevron Osteotomy on Metatarsal Stress: A Finite Element Method

**DOI:** 10.3390/biology11010127

**Published:** 2022-01-13

**Authors:** Qiaolin Zhang, Yan Zhang, Jialu Huang, Ee Chon Teo, Yaodong Gu

**Affiliations:** 1 Faculty of Sports Science, Ningbo University, Ningbo 315211, China; 2011042033@nbu.edu.cn (Q.Z.); zhangyan3@nbu.edu.cn (Y.Z.); hjialu@126.com (J.H.); 2 School of Chemical and Biomedical Engineering, Nanyang Technological University, Singapore 639798, Singapore; 3 Faculty of Engineering, University of Szeged, 6720 Szeged, Hungary

**Keywords:** hallux valgus, metatarsal stress, metatarsophalangeal stress, chevron osteotomy

## Abstract

**Simple Summary:**

In this study, the finite element method was used to explore the effect of distal chevron osteotomy displacement on metatarsal stress. For the subjects used in this study, the metatarsal stress was minimized by moving 4 mm. For the displacement of chevron osteotomy, both postoperative stability and stress distribution of metatarsal should be considered. The most appropriate distance is suggested to be obtained by combining HVA, IMA, age, body weight, and metatarsal width. The finite element method can be used for preoperative estimation of chevron osteotomy and guide the operation.

**Abstract:**

Background: The stress of foot bone can effectively evaluate the functional damage caused by foot deformity and the results of operation. In this study, the finite element method was used to investigate the degree of displacement of distal chevron osteotomy on metatarsal stress and metatarsophalangeal joint load; Methods: Four finite element models of displacement were established by using the CT images of a patient with moderate hallux valgus (hallux valgus angle and intermetatarsal angle were 26.74° and 14.09°, respectively), and the validity of the model was verified. Each finite element model consisted of bones and various cartilage structures, ligaments, and plantar fascia, as well as encapsulated soft tissue. Except for soft tissue, the material properties of other parts were isotropic linear elastic material, and the encapsulated soft tissue was set as nonlinear hyperelastic material. The mesh was tetrahedral mesh. Link elements were used in ligament and plantar fascia. A ground reaction force with a half-body weight was applied at the bottom of the floor to simulate the ground reaction when standing. The upper surfaces of the encapsulated soft tissue, distal tibia, and distal fibula were fixed. The stress distribution of metatarsals and the stress of cartilage of the first metatarsophalangeal joint were compared and analyzed; Results: Compared with the hallux valgus without osteotomy, the stress of the first metatarsals and second metatarsals of 2–4 mm decreased, and the stress of the interarticular cartilage of the first metatarsophalangeal joint with 4 mm was reduced. In the case of 6 mm, the stress value between the first metatarsal and the first metatarsophalangeal joint increased, and 4 mm was the most suitable distance; Conclusions: Compared with the hallux valgus without osteotomy, the stress of the first metatarsals and second metatarsals of 2–4 mm decreased, and the stress of the interarticular cartilage of the first metatarsophalangeal joint with 4 mm was reduced. In the case of 6 mm, the stress value between the first metatarsal and the first metatarsophalangeal joint increased, and 4 mm was the most suitable distance. For the degree of displacement of the distal chevron osteotomy, the postoperative stability and the stress distribution of metatarsal bone should be considered. Factors such as hallux valgus angle, intermetatarsal angle, patient’s age, body weight, and metatarsal width should be considered comprehensively. The factors affecting osteotomy need to be further explored. The degree of displacement of osteotomy can be evaluated by FE method before the operation, and the most suitable distance can be obtained.

## 1. Introduction

Hallux valgus (HV) is a common foot disease [1]. The deformity changes the anatomical structure of the foot, causes biomechanical changes of the foot, affects the weight-bearing of the foot, and presents a variety of clinical symptoms. It is characterized by a progressive lateral deviation of the hallux, accompanied by a medial deviation of the first metatarsal. Hallux valgus angle (HVA) and intermetatarsal angle (IMA) are commonly used radiological indices to evaluate the degree of deformity, which can be divided into mild (HVA: 15–20°; IMA: 9–11°), moderate (HVA: 20–40°; IMA: 11–16°), and severe (HVA: >40°; IMA: >16°) [2]. Increasing HV can lead to major health-related problems, such as metatarsal pain [3], poor balance [4], and an increased risk of falls in the elderly [5].

With the development of the disease, the appearance of the foot of the patients with HV gradually changes. The appearance of the foot is also affected by the accompanying pain and other symptoms. Conservative treatment is the first choice for the treatment of this disease [6]. However, sometimes surgery is needed. There are many treatment methods for HV, and most HV deformities can only be corrected by surgery [7]. It is reported that there are more than 150 surgical methods for HV correction. Barg et al. [8] systematically reviewed studies reporting the results of surgical correction of HV deformities. A total of 229 studies met the inclusion criteria. The results showed that the complication rates of postoperative dissatisfaction and postoperative first metatarsal toe pain were 10.6% and 1.5%, respectively. The total recurrence rate of malformation was 4.9%. Li et al. [9] presented a retrospective analysis of 186 patients who underwent chevron osteotomy combined with distal soft tissue release; 45 patients (24.2%) had a recurrence of HV. The analysis showed that osteotomy was a risk factor for recurrence of HV.

**Figure 1:**
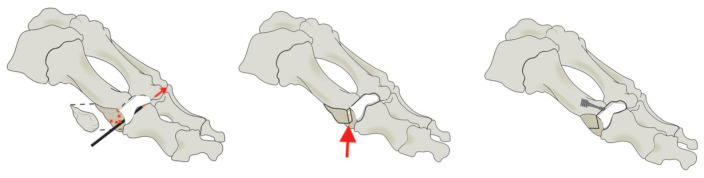
The process of chevron osteotomy. Figure provided by H.J. Trnka.

Chevron osteotomy is distal osteotomy. The method of osteotomy is shown in Figure 1 [10]. As one of the most commonly used methods of HV osteotomy, the clinical effect of chevron osteotomy is satisfactory to both doctors and patients. Vernois and Redfern [11] reported the results of chevron osteotomy for the treatment of 100 feet. Radiological analysis showed that the IMA was corrected from 14.5° to 5.5° at the last follow-up. The average HVA was corrected from 33.7° before operations, to 7.3° at the last follow-up. The satisfaction rate reported by the patients was 95%. How to achieve satisfactory surgical results and, at the same time, relieve pain and prevent complications is a problem worth thinking about by doctors.

In an orthopedic osteotomy, the degree of displacement and shortening of distal osteotomy directly affected the correction effect of the operation. Insufficient displacement cannot effectively reduce the IMA, and excessive displacement is not conducive to the stability of the osteotomy [12]. In clinical treatments, the degree of displacement of distal chevron osteotomy is generally judged according to the X-ray HVA and IMA of the affected foot during distal first metatarsal osteotomy [13]. Timothy M. [14] proposed that, when using chevron osteotomy, the distal metatarsal push should be 50% of the metatarsal shaft width to ensure stability after osteotomy. This study was carried out from the point of view of biomechanics, and the finite element (FE) method was used to explore the influence of different distal metatarsal displacements on the stress distribution of metatarsals. In this study, the influence of the progress of chevron osteotomy on the stress of metatarsal bone was not considered, so the first metatarsal bone was not shortened by modeling.

Computational method using FE analysis provides a versatile platform to evaluate the internal biomechanical environment with controlled and pre-assigned sets of conditions [15,16,17], which was commonly used to understand foot pathology [18], assess the outcome of surgery [19], and design implants [20], etc. The FE method has been widely used in the study of HV. Zhang et al. [21] predicted the metatarsal stress and pressure of a severe HV patient under a balanced standing condition using a comprehensive FE model of the foot and ankle complex. Wong et al. [22] established a musculoskeletal model to explore the effect of ligament relaxation on the etiology of HV. The above studies are all based on the FE method to explore all kinds of foot problems. Simón et al. [23] found that diabetes increased the limitation of ankle movement, which can provide a reference for exploring the effect of stiffness on walking by FE method. Matzaroglou et al. [24] explored the stress difference between 90° chevron osteotomies and 60° chevron osteotomies by FE method. FE method predicted enhanced mechanical bonding, with stronger compressive stresses and weaker shearing stresses with the 90° chevron osteotomy. The influence of different degrees of displacement of chevron osteotomy on the stress of metatarsal bone remained unaddressed. In this study, the FE method was used to establish a series of FE models of HV foot with different degrees of distal metatarsal in chevron osteotomy, to simulate the stress distribution of metatarsal at rest, and to explore the biomechanical properties of HV foot and the biomechanical influence of operation on it. In the past, the evaluation of the curative effect of orthopedic surgery was mostly based on the improvement of clinical symptoms, ignoring mechanical analysis. This study focused on the influence of distal metatarsal extrapolation displacement of chevron osteotomy on metatarsophalangeal joint stress, from the point of view of biomechanical mechanism, and made a comparative analysis to provide a theoretical basis for selecting the best operation plan in clinical treatment.

## 2. Materials and Methods

### 2.1. Data Acquisition

**Figure 2:**
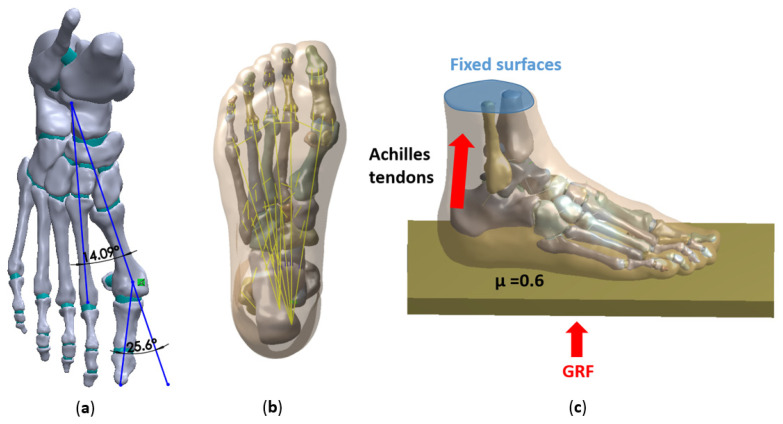
(**a**) HIA and IMA in patients with moderate HV; (**b**) Plantar fascia and ligaments; (**c**) Boundary conditions and load.

In this study, a female patient with moderate HV (age: 30 years old; height: 165 cm; weight: 50 kg) was selected. Her HVA and IMA were 26.74° and 14.09°, respectively, as shown in Figure 2a. A three-dimensional model of the HV foot was reconstructed by CT. In the past 12 months, the participants had no other musculoskeletal pathology, pain, lower limb injury, or surgery. The study was approved by the school ethics committee (ARGH20211115).

### 2.2. Model Construction

The right foot of the subject was scanned by CT at intervals of 2 mm, and the right foot was standing at the time of shooting. Mimics16.0 (Materialise, Leuven, Belgium) was used to segment the two-dimensional image, and the three-dimensional model of bone tissue and capsule soft tissue was obtained, using Geomagic Studio 2013 (Geomagic, Inc., Research Triangle Park, NC, United States) to smooth the uneven model. Each surface component was then imported into SolidWorks 2020 (SolidWorks Corporation, Waltham, MA, United States) to form solid parts. 

To model the cartilage structure, a solid was created between the adjacent surfaces of two bones. All bones and cartilage from all soft tissue were subtracted to form encapsulated soft tissue. The foot numerical model consisted of 30 bone segments, including tibia, fibula, talus, calcaneus, cuboid bone, scaphoid bone, 2 sesamoid bone, 3 cuneiform bone, 5 metatarsal bone, and 14 phalanges. The connection unit, with tension-only capacity, was used to simulate ligaments. A total of 76 ligaments and 5 plantar fasciae were included, and the attachment points on the corresponding bones were connected by a straight line structure, as shown in Figure 2b. The attachment area was determined by referring to the anatomy book [25], and the attachment point was close to the geometric center of the attachment area. The metatarsal bone was cut in SolidWorks to simulate the models of different degrees of chevron osteotomy. The distances were 0 mm, 2 mm, 4 mm, and 6 mm, and the chevron osteotomy angle of each model was 90° [24].

The four models were imported into Workbench19.2 (ANSYS, Inc., Canonsburg, PA, USA) for meshing and contact settings. All solid parts were divided by tetrahedral meshes. Based on the age matching model, which passed the mesh convergence test before, the mesh sizes of encapsulated soft tissue, bone, and cartilage were set to 3 mm, 2 mm, and 0.5 mm, respectively. In addition, local refinement was carried out to adapt to the geometric shape of the contact area. 

Workbench provided automatic contact detection for parts. Using an algorithm based on surface proximity, you can create possible contact pairs. Face-to-face contact was used to simulate the interaction between cartilage and bone surface. The contact between the surface of bone and cartilage was set to frictionless [26]. All the bones and cartilage were glued to the encapsulated soft tissue. The interaction between the foot and the plate was simulated as a contact surface with a friction coefficient of 0.6 [27]. The rest of the contracts were set to bind.

### 2.3. Boundary, Loading Conditions

The FE method was used to analyze the state of the right foot standing in balance. The upper surface of the soft tissue, the distal tibia, and the distal fibula were fixed as shown. A plate with elastic properties was created to simulate the ground support. The board can only move freely in the vertical direction. The vertical ground reaction force of the half-body weight was applied to the lower surface of the plate 250 N. The interaction between the foot and the plate was simulated as a contact surface with a friction coefficient of 0.6 [27]. An equivalent force vector 125 N [28], representing the force of the Achilles tendon, was applied at the posterior end of the calcaneus, as shown in Figure 2c.

Except for the encapsulated soft tissue, all materials were regarded as isotropic and linear elastic materials, and their properties were obtained from previous literature [29,30,31,32,33]. Two material constants, Young’s modulus (E) and Poisson’s ratio (v), were specified to represent elasticity. The encapsulated soft tissue was set as a nonlinear hyperelastic material, which was defined as the Moonley–Rivlin model. The material properties of each component are listed in Table 1.

### 2.4. Validation of the FE Models

The verification was verified by comparing the plantar pressure measured in the experiment with the finite element prediction in the standing stage without osteotomy. The plantar pressure of the same subject was measured by the Emed plantar pressure plate (Novel, Munich, Germany). The subjects were asked to stand still on the Emed pressure plate for 5 s [21], and the peak contact pressure and pressure distribution were recorded. The peak pressure and pressure distribution measured by the experiment were compared with the data predicted by the finite element method to verify the effectiveness of the model.

## 3. Results

### 3.1. Model Building

**Figure 3:**
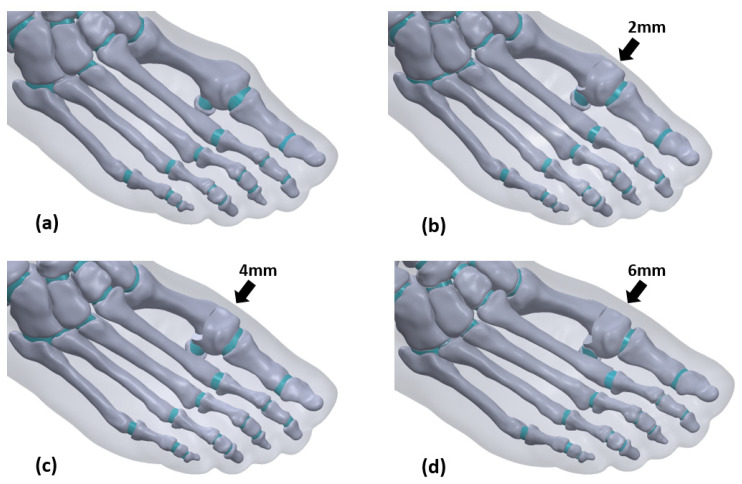
(**a**–**d**) Four models of distal metatarsal displacement were performed with no osteotomy, and the displacement of osteotomy was 2 mm, 4 mm, and 6 mm.

In this study, a total of four models were established, namely the moderate HV right foot without osteotomy and the model chevron osteotomy passing 2 mm, 4 mm, and 6 mm, as shown in Figure 3. After osteotomy, the number of units, HVA, and IMA are shown in Table 2.

### 3.2. Verification Result

**Figure 4:**
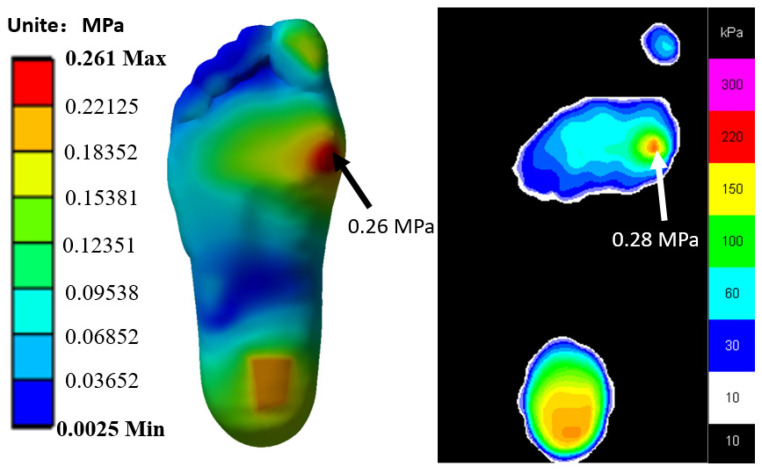
Comparison of the plantar pressure between computational prediction and experimental measurement in a balanced standing position.

The plantar pressure predicted by FE method was compared with that obtained by experiment. The results show that the data are in good agreement with each other. From the results, when standing in balance, the plantar pressure was mainly concentrated in the heel and the inside of the forefoot, as shown in Figure 4. The peak values of plantar pressure are all on the inside of the forefoot, and the peak data measured by FE simulation and experiment were 0.26 MPa and 0.28 MPa, respectively, with a difference of only 7.6%, which proves that the model is effective.

### 3.3. The Stress of the Metatarsals

**Figure 5:**
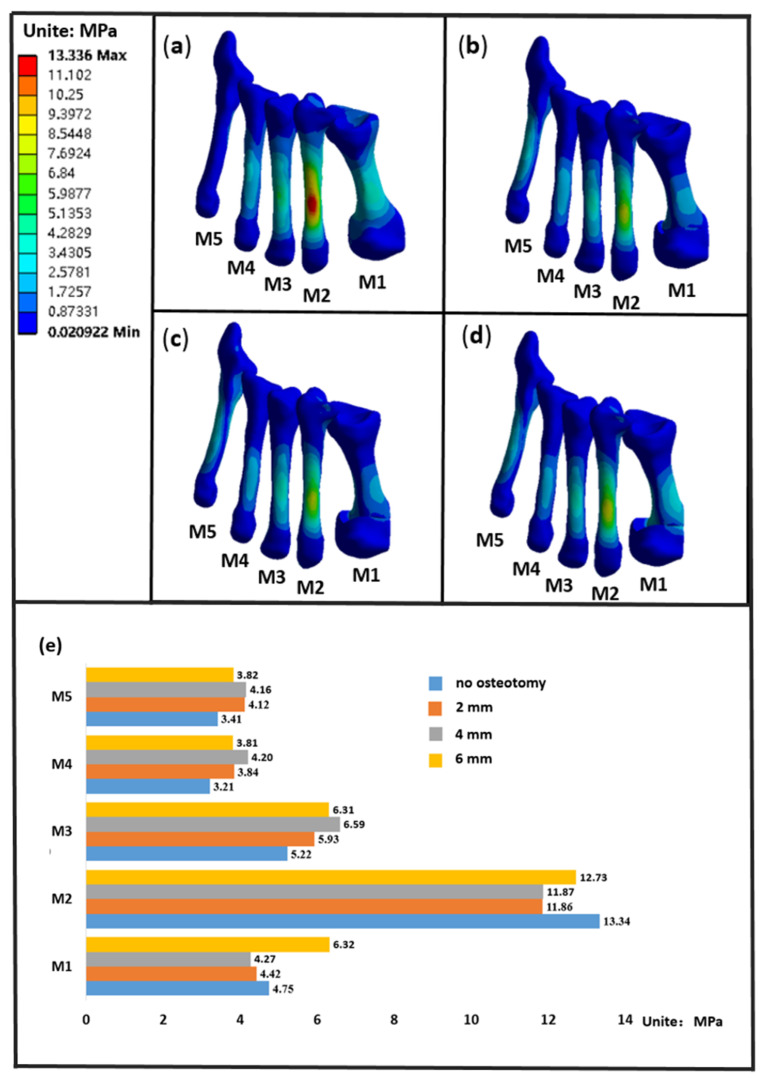
(**a**–**d**) is the stress distribution of metatarsal bone no osteotomy, 2 mm, 4 mm, 6 mm, respectively, (**e**) is the maximum stress of each metatarsal. M1—M5 is the first to fifth metatarsals, respectively.

Among the five metatarsals, M2 had the highest stress, followed by M3 and M1, followed by M4 and M5. The stress distribution and stress value of metatarsals are shown in Figure 5. In Figure 5a,b, it can be seen that there is obvious stress concentration in M2 without osteotomy, which can effectively slow down the problem of stress concentration in M2 after osteotomy. From the maximum stress value of each metatarsal in Figure 5e, it can be seen that the stress of the first metatarsal can be effectively reduced when the displacement was 2 mm and 4 mm; the stress of the first metatarsal was reduced by 6.9% and 10.1% respectively, while, in the case of 6 mm, the stress of the first metatarsal increased by 33.1%. Compared with the model without osteotomy, the stress of M3, M4, and M5 increased after osteotomy. After osteotomy, the overall stress of the foot transferred to the displacement side (the lateral side of the foot).

### 3.4. The Stress of the First Metatarsophalangeal Articular Cartilage

**Figure 6:**
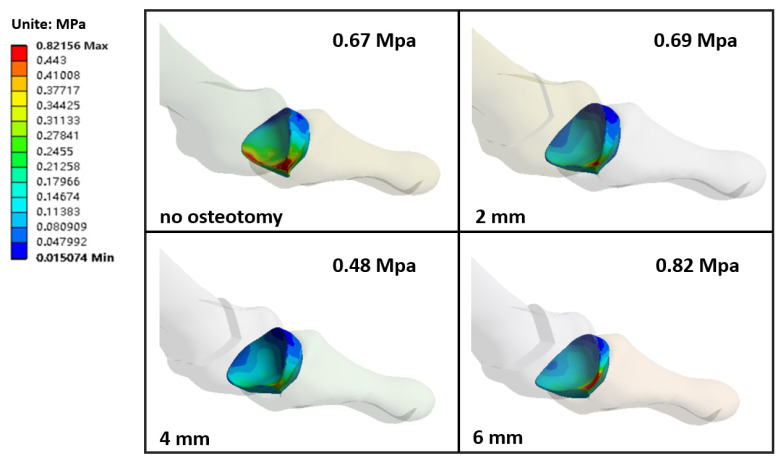
Stress distribution and stress value of the first metatarsophalangeal articular cartilage.

There is cartilage between metatarsals and phalanges, and the cartilage between metatarsophalangeal joints is defined as joint stress. The stress value of the first metatarsophalangeal joint of each model is shown in the Figure 6. The stress value of the first metatarsophalangeal articular cartilage of 4 mm was significantly lower than that of no osteotomy. Compared with no osteotomy, the stress of the first metatarsophalangeal articular cartilage of the 6 mm model and metatarsophalangeal joint increased by 3.1% and 22.7%, respectively. From the stress distribution map of cartilage, it can be seen that the stress of no osteotomy cartilage was concentrated in the lower part of the cartilage. After osteotomy, the stress distribution on the inner and outer side of cartilage was asymmetrical, especially 2 mm and 6 mm, and the stress was mainly concentrated in the lower part. 

## 4. Discussion

In this study, the HV model of the right foot was established by using the FE method. The model was assembled and modified in SolidWorks to simulate the models of different degrees of chevron osteotomy, and the stress of the metatarsal was analyzed from the point of view of biomechanics. In the past, the curative effect evaluation of orthopedic surgery was based on the improvement of clinical symptoms, ignoring the mechanical analysis. The FE analysis method uses the approximate mathematical method to divide the physical system into many small elements for simulation, which can simulate and calculate the structures with complex geometric shapes and material properties [34]. The FE method has a powerful modeling function, and it can simulate objects with complex geometric shapes, material parameters, and different stress conditions under static and dynamic conditions [35]. It can not only repeat and change various stress areas and directions for complex stress analysis, but also keep the physical properties of the model constant, which can make up for the shortcomings of in vitro cadaveric experiments and animal experiments. It is widely used in biomechanical research [36,37]. This study has shown that the finite element method can well explore chevron osteotomy, to explore the effect of different degrees of extrapolation on metatarsal stress. The FE method can be used not only to evaluate the mode of operation, but also to evaluate the effect of different implantation methods of internal fixation instruments. The study of López et al. [38] showed that in different foot diseases, it was found that the optimal level of fixation of different types of Lapidus plate systems in foot surgery was related to the type of screw plate and the implant location of the system. The number of studies on the effectiveness of Lapidus plate systems in foot surgery is insufficient, and it is necessary to increase the knowledge of the results of fixation level, system type, and insertion position in foot surgery. The FE method can effectively explore this problem, and it can also explore the influence of a muscle on the biomechanics of the foot through the musculoskeletal system combined with FE method [39]. Palomo et al. [40] found that the existence of fibularis tertius muscle was variable and was not affected by individual sex. This study can provide a good idea for further research. In recent years, FE analysis has been widely used in the field of foot and ankle surgery, which provides strong support for clinical research on the etiology, pathological mechanism, treatment, and rehabilitation intervention of foot diseases [37]. In this study, from the point of view of three-dimensional mechanics, a good therapeutic effect should not only correct the deformity, but also restore near-normal plantar pressure distribution and prevent recurrence and other complications.

During the orthopedic osteotomy, the degree of displacement directly affected the operation’s correction effect, which was not enough to reduce the IMA effectively; too much to the outside was not conducive to the stability of the osteotomy end [41]. In clinical treatment, the degree of distal metatarsal moving outward after osteotomy is generally judged according to the HVA angle and IMA angle of the X-ray taken of the affected foot [13]. In chevron osteotomy, different scholars hold different views on this, and the degree of distal metatarsal displacement is also different. The chevron osteotomy of the distal first metatarsal was described by Corless [42] in 1976 as a modification of the Mitchell procedure for the correction of bunions associated with mild to moderate metatarsus primus varus. Some scholars have reported the results of using chevron osteotomy [43,44]. They suggested that the lateral displacement of the metatarsal head should be only 2–4 mm to prevent it from falling off from the side. Austin et al. [45], reported the experience of chevron osteotomy in 1981. They describe shifting the metatarsal head to 1/4 to 1/2 of the width of the metatarsal shaft to correct varus. Other authors have also proposed guidelines for the lateral displacement of the metatarsal head [46,47,48]. Timothy M. et al. [14] proposed that the distal metatarsal distal displacement should be 50% of the metatarsal shaft width when using chevron osteotomy, to ensure stability after osteotomy.

The above research focuses on the stability of metatarsals after osteotomy and does not analyze the stress of metatarsals and metatarsophalangeal joints. In this study, the metatarsal width of the subject was 12.2 mm. Since the problem of postoperative stability was not considered in this study, the contact between bones after osteotomy was set to “bond”. The study showed that when the width of the metatarsal was pushed to 50%, that is, when the foot was moved 6 mm, the stress of other metatarsal bones except the second metatarsal bone was significantly increased compared with that of the foot without osteotomy; the stress of the first metatarsal bone was increased by 33.1%. Moving 6 mm was the most stable, but not the most suitable. Zhang et al. [21] showed that the stress value of the first metatarsal of the bunion foot was greater than that of a normal foot. Chevron osteotomy is not only used to improve the appearance of the foot, but also to reduce stress on the abnormal metatarsal of the foot with HV [49]. In the case of 2 mm and 4 mm displacements, the stress of the first metatarsal decreased by 6.9% and 10.1%, respectively. The stress of the second metatarsal also decreased, but the stress of the third, fourth, and fifth metatarsal increased, and the stress of the first and second metatarsal gradually transferred to the lateral after osteotomy. Based on these results, displacement of 2 mm and 4 mm, compared to 6 mm (50% of the metatarsal shaft), can reduce the stress of the first metatarsal and avoid complications such as pain in the corpus callosum.

In this study, the cartilage in the middle of the metatarsophalangeal joint represents the stress of the metatarsophalangeal joint. It was found that the stress distribution of the first metatarsophalangeal articular cartilage of the HV foot without osteotomy was mainly concentrated near the lateral base of the second metatarsal bone, and there was also stress concentration at the medial bottom. After the operation, the stress concentration at the medial bottom disappeared, and 2 mm had little effect on the stress at the lateral base. However, after moving 6 mm, the stress of the first metatarsophalangeal articular cartilage increased by 22.4%, which may aggravate the symptom of “painful bunion” which is one of the most common complaints among HV patients [50]. The stress of the first metatarsophalangeal joint decreased only when the 4 mm was moved. According to the above results, 4 mm was the most suitable for patients with moderate HV, with an HVA of 25.6° and an IMA of 14.1°. The push amount of chevron osteotomy should be considered based on the stability and the stress distribution of metatarsals after operation. As a result, a combination of HVA, IMA, age, weight, and width of metatarsals of patients [14] is essential for obtaining the most suitable distance. However, the factors that affect the effect of surgery are not limited to the above factors.

As an important part of digital medicine, FE analysis will play a greater potential in the study of HV deformity [51]. Through the analysis of the FE model of HV foot, we can fully understand the various surgical treatment methods and the long-term biomechanical changes after operation, which has important clinical significance for predicting the curative effect of operation and the prevention of complications [52,53]. However, in the FE analysis method, the establishment of the model approximately simulates the organism by the way of digital modeling. In this study, the ligament properties of the operated and unoperated models were the same, and the effect of operation on the ligament was not taken into account. A greater or lesser rigidity of the plantar structures (ligaments mainly) and how these structures influence the mobility of the first radius were not contemplated, alongside how this can affect the stress distribution of metatarsals and the stress of cartilage of the first metatarsophalangeal joint. In terms of materials, except for the encapsulated soft tissue, all materials were regarded as isotropic and linear elastic materials. The bone was divided into cortical bone and cancellous bone. If the bone was defined as a linear elastic material, the stress value of the bone would increase, which needs to simplify some secondary organization and structure of the complex organism, which cannot be completely accurate. In addition, the results were based on some assumptions due to the use of FE, that may be reflected as a potential limitation. Therefore, improving the geometric similarity and accuracy of FE model is an important direction of FE analysis in biomechanics research.

## 5. Conclusions

Four FE models were established, simulating different degrees of displacement of chevron osteotomy, to predict the internal stress of metatarsals and the loading of metatarsophalangeal joints during balanced standing. Displacement of 4 mm was the most suitable for patients with moderate HV, with an HVA of 25.6° and an IMA of 14.1°. For the displacement of chevron osteotomy, both postoperative stability and stress distribution of metatarsals should be considered. The most appropriate distance was suggested to be obtained by combining HVA, IMA, age, body weight, and metatarsal width. The factors affecting osteotomy need to be further explored. The material properties of ligament, cortical bone, and cancellous bone of the model will be further improved, and the effect of muscle strength on the foot model will be considered, to obtain more accurate prediction and better comparison.

## Figures and Tables

**Table 1:** Material parameters of foot FE model.

Component	Yang’s Modulus E (MPa)	Poisson’s Ratio (v)	Cross-Section Area (mm^2^)
Bone	7300	0.3	
Cartilage	1	0.4	
Ligament	260	0.4	18.4
Plantar Fascia	350	0.4	58.6
Plate	17,000	0.4	

**Table 2:** The number of units and HVA, IMA.

	HVA	IMA	Nodes	Elements
no osteotomy	25.6°	14.1°	2061317	1391011
2 mm	20.3°	11.3°	2059307	1389514
4 mm	15.4°	9.3°	2059749	1390233
6 mm	11.6°	8.4°	2055713	1387394

## Data Availability

The data that support the findings of this study are available on reasonable request from the corresponding author. The date is not publicly available, due to privacy or ethical restrictions.
